# Supplementary material for: The Lacto-Tetrapeptide Gly–Thr–Trp–Tyr, β-Lactolin, Improves Spatial Memory Functions via Dopamine Release and D1 Receptor Activation in the Hippocampus
Source: Nutrients. 2019 Oct 15;11(10):2469. doi: 10.3390/nu11102469 (PMC6835598; doi:10.3390/nu11102469)
Supplement: Supplementary file 1 [file nutrients-11-02469-s001.pdf]

Supplementary Information

The lacto-tetrapeptide Gly–Thr–Trp–Tyr, β-lactolin, improves spatial memory functions via dopamine release and D1 receptor activation in the hippocampus

Authors

Tatsuhiro Ayabe[1]*, Yasuhisa Ano[1], Rena Ohya[1], Shiho Kitaoka[2, 3], Tomoyuki Furuyashiki[2, 3]

Address

[1] Research Laboratories for Health Science & Food Technologies, Kirin Holdings Company Ltd, 1-13-5 Fukuura Kanazawa-ku, Yokohama-shi, Kanagawa 236-0004, Japan

[2] Division of Pharmacology, Kobe University Graduate School of Medicine, Kobe, Japan

[3]AMED-CREST, Chiyoda-ku, Tokyo 100-0004, Japan.

***Corresponding Author**

Tatsuhiro Ayabe, Research Laboratories for Health Science & Food Technologies, Kirin Holdings Company Ltd., Yokohama, Kanagawa, 236-0004, Japan; Tel.: +81-45-330-9007, Fax.: +81-45-782-3657. E-mail: Tatsuhiro_Ayabe@kirin.co.jp

Supplementary procedures

Morris water maze test

The apparatus used in this study was a circular pool (120 cm in diameter) filled with water. A transparent escape platform (12 cm in diameter) was placed at the center of one of the pool's quadrants, submerged 1.5 cm below the water surface. Prior to the test, the mice were released into an equilateral triangular pool (60 cm of side) with a platform, to acclimatize to both the water and the platform. During the training period, the mice were released into the pool facing the wall from each quadrant in a random order. Each trial ended either when the mouse climbed onto the platform or when it failed to find the platform within 60 s. Each trial was repeated four times per day, with 30 s inter-trial intervals. The training was performed for 5 consecutive days, after which the mice were subjected to the probe test. In the probe test, the mice were released into the pool in the absence of the platform, and allowed to explore for 60 s. The latency to the target (i.e., the place where the platform used to be) and the time spent in the target quadrant were measured. In this study, the mice were tracked using the SMART 3.0 system (Panlab, Barcelona, Spain) by means of a digital video camera mounted on the ceiling.

Supplementary Fig. 1

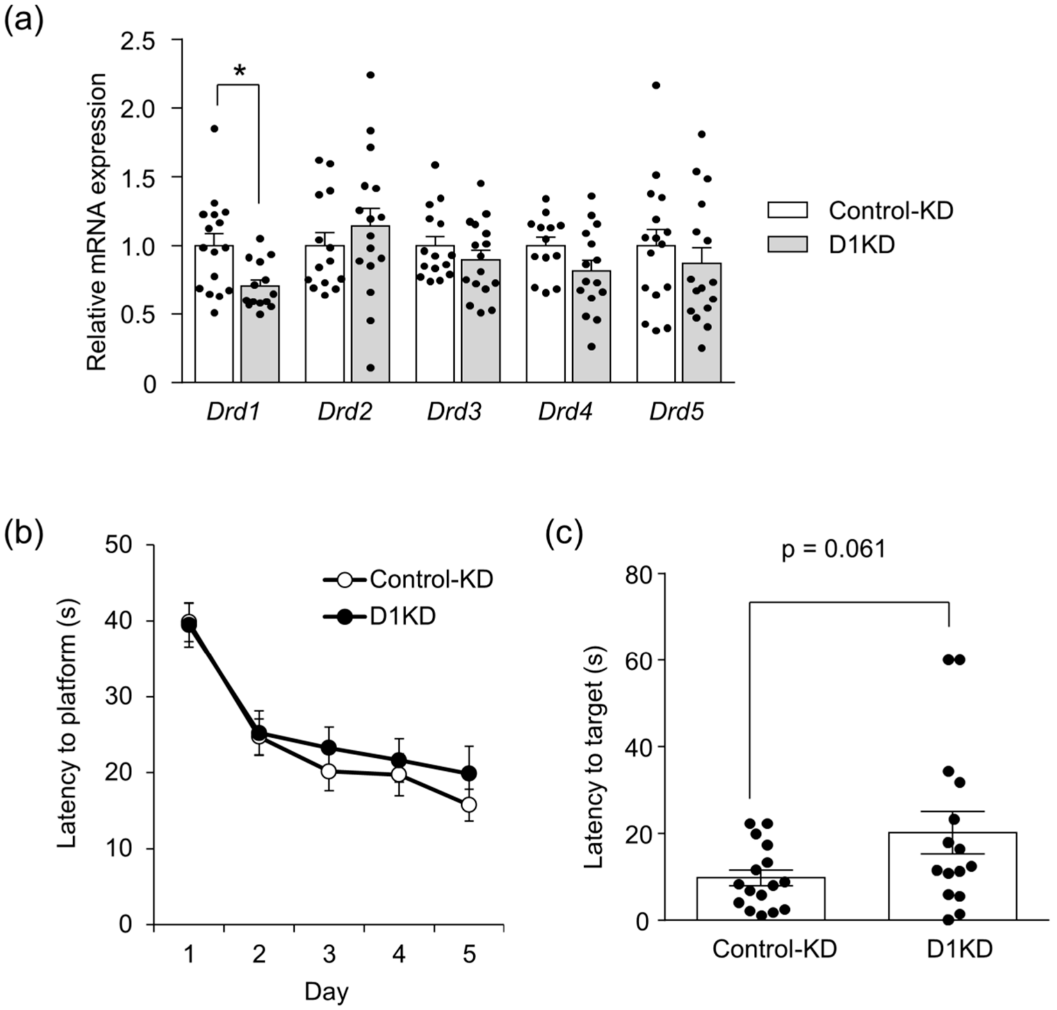

Supplementary Figure 1. Effects of the hippocampal D1 receptor knockdown on spatial reference memory in the Morris water maze.

(a) Expression of hippocampal DA receptor subtype mRNA in knockdown mice or control-knockdown mice. Data are normalized by the expression of GAPDH, and expressed relative to control-knockdown mice. (b, c) The spatial learning and memory functions of hippocampal DA D1 knockdown mice and control-knockdown mice were evaluated using the Morris water maze. (b) Latency to platform during the training period. (c) Latency to target zone and time spent in the target quadrant during the probe test, respectively. Results are presented as the mean ± SEM (n = 16 mice per group). *$p < 0.05$ *versus* the control-knockdown group.
